# The association between social capital and quality of life in old adults: a systematic review and meta-analysis

**DOI:** 10.3389/fpubh.2025.1668696

**Published:** 2025-11-06

**Authors:** Alessandra Buja, Sohail Akhtar

**Affiliations:** 1 Department of Cardiological, Thoracic and Vascular Sciences and Public Health, University of Padova, Padova, Italy; 2 Department of Statistical Sciences, University of Padova, Padova, Italy

**Keywords:** social capital, quality of life, older adults, systematic review, meta-analysis

## Abstract

**Background:**

Life satisfaction and quality of life are essential indicators of wellbeing in older adults. Social capital has been increasingly recognized as a key factor influencing these outcomes. This study systematically reviewed and synthesized existing evidence on the association between social capital and quality of life and life satisfaction among older adults through a systematic review and meta-analysis.

**Methods:**

A comprehensive literature search was performed in MEDLINE (via PubMed), PsycINFO, and CINAHL (via EBSCO) from inception to January 15, 2025. Observational studies reporting quantitative associations between social capital and quality of life and life satisfaction in adults aged ≥60 years were included. Unadjusted effect sizes (*r*) were pooled using random-effects models for meta-analysis to account for variability across studies. Subgroup meta-analyses were conducted to examine differences based on publication period, geographic location, and quality of life measures. Between-study heterogeneity was tested using the *I*^2^ index, and publication bias was investigated using funnel plots, Egger's test, and Begg's test.

**Results:**

We identified 13 studies that included 5,880 older participants from seven countries. Meta-analyses revealed life satisfaction (*r* = 0.25, 95% CI: 0.20–0.31) and quality of life (*r* = 0.35, 95% CI: 0.19–0.49) all demonstrated significant associations with social capital. The overall pooled effect size (*r* = 0.27, 95% CI: 0.22–0.32) demonstrated a consistent positive relationship. Subgroup analyses showed that cognitive social capital (*r* = 0.35, 95% CI: 0.18–0.49) had a stronger association than structural social capital (*r* = 0.24, 95% CI: 0.19–0.29). Regional differences were not statistically significant (*p* = 0.182), although the effect sizes varied across continents: America (*r* = 0.24, 95% CI: 0.16–0.32), and Asia (*r* = 0.30, 95% CI: 0.23–0.37). Statistical heterogeneity was observed across meta-analyses (*I*^2^ = 68.9–95.5%). Publication bias was not significant based on Egger's and Begg's tests.

**Conclusions:**

The findings of this meta-analysis suggest that social capital, particularly its cognitive dimension, plays an important role in enhancing quality of life and life satisfaction outcomes, with differences across time and geographic regions.

**Systematic review registration:**

https://www.crd.york.ac.uk/PROSPERO/view/CRD42025638236, identifier: CRD42025638236.

## Introduction

Population aging is rapidly reshaping global demographics, emerging as one of the major trends of the 21st century. Individuals worldwide are experiencing longer lifespans with most expecting to live into their mid-sixties and beyond (1). Countries across all income levels are reporting growth in both the number and percentage of older adults in their populations (2). The population of individuals aged 60 or older is projected to rise from 1 billion in 2020 to 1.4 billion by 2030, representing approximately one in every six adults. By 2050, the number of older adults is projected to double to 2.1 billion, while those aged 80 and above are forecasted to triple, reaching 426 million (2). Population aging first emerged in high-income countries, like Japan, where around 28% of the population is above 65. According to WHO report (2), over 66% of people aged 60 and older will be living in these regions by 2050. However, the most significant changes are now occurring in low- and middle-income countries (3). These demographic shifts are creating complex challenges for communities globally, involving healthcare and caregiving, regardless of a nation's cultural, economic, or political structure (4). Rising life expectancy poses challenges for healthy aging, increasing vulnerability among older adults. A strong community social environment enhances healthy aging by reinforcing social capital.

Social capital, defined by the networks, relationships, social norms, and social trust that facilitate collective action and collaboration, is of considerable significance for an aging population (5). People with strong social connections have improved physical and mental health, including lower risk of depression, loneliness, and cognitive decline, and better quality of life (5–7). Social connections motivate older people to actively participate in their communities and lead healthy lifestyles because they provide a sense of purpose and belonging (8). Individuals who feel supported by their relationships, such as family, friends, and community members are more likely to maintain good mental health, cope effectively with challenges, and experience overall life satisfaction (9). Generally, social capital can be classified as structural social capital, that is, the observable features of social networks that is, belonging to organizations and community groups, or cognitive social capital, the composite of trust, reciprocity, and shared values (10). Structural social capital provides pathways to essential resources, medical care, and community involvement, while cognitive social capital strengthens emotional resilience and interpersonal trust (10). More broadly, social capital is thought to provide a better quality of life and resilience in older adults; therefore, the need for building social networks in communities dealing with an aging population is imperative (11). However, existing studies show inconsistent results due to variations in context, measurement of social capital, and study design. Recent research indicates that there are typically higher levels of quality of life among older adults who are socially engaged, trust people, and have network support, but results are mixed across different socioeconomic, cultural, or health contexts (12–14). While there is an increasing interest in these community forces, the existing literature still has not fully presented overview on the current state of quality of life of aged population nor incorporated a range of academic perspectives. This meta-analysis addresses these gaps by providing a comprehensive and quantitative synthesis of the association between social capital and quality of life in older adults. This review aims to synthesize global evidence on the association between social capital (structural and cognitive) and quality of life and life satisfaction among older adults.

## Methods

The review protocol was preregistered in the International Prospective Register of Systematic Reviews (PROSPERO; CRD42025638236) to ensure transparency and minimize reporting bias. The review followed the Preferred Reporting Items for Systematic Reviews and Meta-Analyses (PRISMA) guidelines (15) for evidence identification, screening, and synthesis (see in Supplementary Table S2).

### Search strategy

A thorough search was carried out across three major databases: Medline (PubMed), CINAHL, and PsycINFO, from their inception up to January 15, 2025. Search terms combined concepts related to older adults, social capital, and outcomes of interest. Boolean operators were applied to refine the strategy. The specific search strings utilized are provided in Supplementary Table S1. Upon retrieval, the records were imported into Zotero software (version 7) for management, where duplicates were systematically detected and eliminated. Additionally, the reference lists of the selected studies were scrutinized to detect any potentially relevant articles that might have been missed in the primary database screening.

### Inclusion criteria

Articles were included if they met all of the following criterias:

1. Participants were aged 60 years or older.
2. The study employed an observational design (cross-sectional, cohort, or case-control).
3. The article was written in English.
4. The study investigated quantitatively the association between social capital and quality of life or life satisfaction among older populations.

### Exclusion criteria

Articles were excluded if they met any of the following criteria:

1. Publications such as literature reviews, meta-analyses, case reports, editorials, commentaries, theses, or book chapters.
2. Studies that involved only patients with specific medical conditions.

### Data extraction

Data extraction was performed utilizing a customized Microsoft Excel spreadsheet developed in line with the aims of our review. Two authors independently extracted key study characteristics, including the first author's surname, year of publication, survey year and country of origin, participant demographics, such as age range, mean age, gender distribution, sample size, and effect sizes, specifically indicators of social capital and quality of life metrics. Discrepancies were addressed through discussion between two authors (S.A and A.B.).

### Risk of bias assessment

The risk of bias in the included studies was evaluated using the modified Newcastle-Ottawa Scale (NOS) for observational studies (16). Two authors (S.A. and A.B.) independently performed the risk of bias assessment. Examining three important domains—participant selection, comparability, and outcome evaluation, the scale offers a methodical structure. Studies were graded as low (≥8 points), moderate (5–7 points), or high (< 5 points) risk of bias (16). Discrepancies between the authors were resolved through consensus to settle differences in ratings.

### Data synthesis

All statistical analyses were performed using R, version 4.4.2 (RStudio) with the support of the “meta” package (version 8.1-0) and “metafor” package (version 4.8-0). A random-effects meta-analysis was utilized to pool the effect sizes across studies, thus assuring the results are generalizable to a wider population. For the meta-analysis, we selected the Pearson *r* correlation coefficient as the effect size to represent the association between social capital and quality of life and life satisfaction. All outcomes were transformed into Fisher *z* values and subsequently converted back to their original scale for reporting the pooled results (17). The outcome measurement was the correlation coefficient (*r*), with 95% confidence intervals displayed in a forest plot. Pooled effect sizes were characterized according to the McGrath and Meyer (18) framework as high (*r* ≥ 0.37), moderate (0.10 < *r* < 0.37), or weak (*r* ≤ 0.10). Study heterogeneity was quantified using *I*^2^ values and tested for statistical significance with Cochran's *Q* test (19). A funnel plot offered a preliminary qualitative evaluation, whereas quantitative analyses, such as the Begg's and Egger's tests (20, 21) were employed to measure potential bias. Subgroup meta-analyses were performed to investigate variations in effect sizes based on key categorical variables. The parameters included components of social capital (structural and cognitive), publication timeframes (before 2015 and from 2015 onward), and geographic locations (America and Asia). Univariable meta-regression analyses were performed to investigate the impact of possible continuous mediators on the observed heterogeneity across studies. The mediators comprised mean age, methodological quality, survey year, publication year, male proportion, and sample size. *R*^2^ values were computed to measure the extent of heterogeneity explained by the incorporated modifiers. The robustness of our findings was tested through sensitivity analyses, where each study was systematically excluded, and the pooled effect size was recomputed (22). The findings were deemed statistically significant at *p* < 0.05. All significance tests were two-sided.

## Results

**Figure 1:**
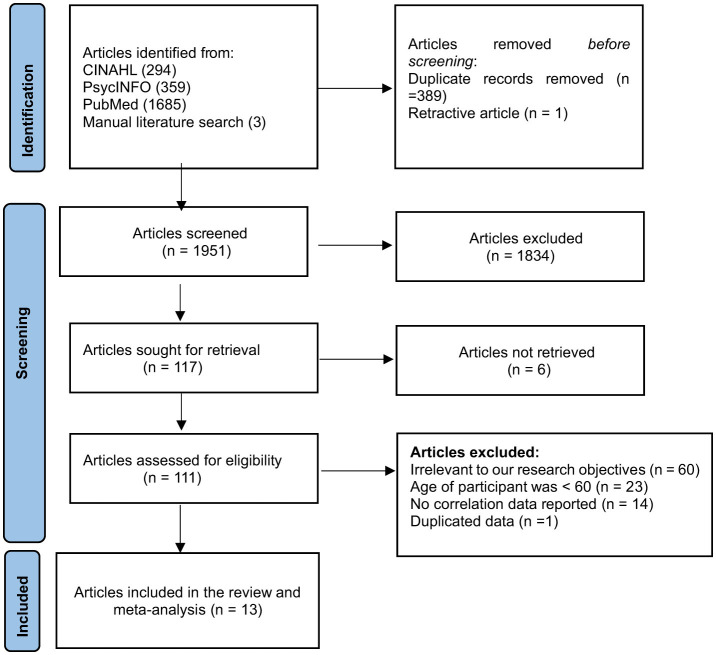
Study selection (PRISMA) flowchart of the association between social capital and quality of life.

A total of 2,341 records (Figure 1) were identified (PubMed: 1,685; CINAHL: 294; PsycINFO: 359; manual literature search: three). After removing 389 duplicates and one retracted article, 1,951 records remained for screening. Of these, 1,834 were excluded based on titles and abstracts, leaving 117 full-text reports for review. Six could not be retrieved, and 98 were excluded for various reasons (irrelevance, age < 60, missing data, or duplication). Ultimately, 13 studies met the inclusion criteria and were included in the review and meta-analysis.

### Study characteristics

A detailed overview of key study characteristics is provided in Table 1. The sample comprised 13 studies (23–35), all published between 1983 (29) and 2023 (24), with a significant portion published recently (77% of the research studies were published after 2010). Sample sizes ranged from 35 (23) to 1,280 (24) participants. The average weighted age of the samples in the review was 73.4 years. Seven effect sizes focused on the association of social capital with quality of life, while 27 focused on life satisfaction. In the included studies, a variety of indicators were used to measure social capital. These indicators encompassed both interpersonal and community-level constructs such as social support, trust in the community, social norms, participation in community activities, and civic engagement. To enable comparability across studies, we classified these diverse measures into two overarching domains: cognitive social capital (24%) and structural social capital (71%). Cognitive social capital consists of several elements, including perceived trust, social support, and shared norms. Structural social capital incorporates observable behaviors and interactions, such as social participation, network ties, group activities, and participation in civic or political activities. Four (31%) studies were conducted in the USA, 3 (23%) in Iran, 2 (15%) in China while one (7%) each in Canada, Hong Kong, South Korea, and Japan. The risk of bias analysis based on NOS indicated that 12 (92%) studies were classified as having a low risk of bias, while 1 (8%) study exhibited a moderate risk of bias. The comprehensive evaluation findings are displayed in Supplementary Table S2.

**Table 1:** Key features of studies in the meta-analysis.

**Author**	**Publication year**	**Country**	**Sample size**	**Age range (year)**	**Average age (year)**	**Design**	**Effect size**	**Risk of bias**
Amiri et al. (23)	2017	Iran	35	65–74	73.5	Cross-sectional	Life satisfaction × social participation, *r* = 0.263	Moderate
Oh and Bae (24)	2023	Korea	1,280	60–79	66.83	Cross-sectional	Life satisfaction × structural social capital, *r* = 0.215	Low
							Life satisfaction × cognitive social capital, *r* = 0.573	
Usui et al. (25)	1985	USA	643	≥60	70	Cross-sectional	Life satisfaction × visit relative, *r* = 0.14	Low
							Life satisfaction × visit neighbor, *r* = 0.21	
							Life satisfaction × visit friend, *r* = 0.24	
Fukuzawa and Sugawara (26)	2023	Japan	418	≥75	79.06	Cross-sectional	Life satisfaction × social support, *r* = 0.06	Low
							Life satisfaction × social participation, *r* = 0.21	
Kim et al. (27)	2015	USA	172	≥65	78.8	Cross-sectional	Life satisfaction × trust on community, *r*= 0.07	Low
							Life satisfaction × participation in community, *r*= 0.19	
			205	≥65	73.8		Life satisfaction × trust on community, *r* = 0.27	
							Life satisfaction × participation in community, *r* = 0.14	
Atri et al. (28)	2020	Iran	522	≥60	65.7	Cross-sectional	Quality of life × social capital, *r* = 0.4	Low
Deimling et al. (29)	1983	USA	129	≥73	73	Cross-sectional	Life satisfaction × group activity, *r* = 0.12	Low
							Life satisfaction × social resources, *r* = 0.11	
							Life satisfaction × group activity, *r* = 0.21	
							Life satisfaction × social resources, *r* = 0.05	
Kim et al. (30)	2019	USA	205	≥65	73.79	Cross-sectional	Quality of life × social norms, *r* = 0.49	Low
							Quality of life × social trust, *r* = 0.4	
							Quality of life × partnership with the community, *r* = 0.48	
							Quality of life × information sharing, *r* =0.39	
							Quality of life × political participation, *r* =0.02	
Wang et al. (31)	2022	China	245	≥80	84.43	Cross-sectional	Life satisfaction × social capital, *r* = 0.451	Low
Levasseur et al. (32)	2010	Canada	156	≥60	73.7	Cross-sectional	Life satisfaction × participation in social roles, *r* = 0.4	Low
Fu et al. (33)	2023	China	950	≥60	70.5	Cross-sectional	Quality of life × social capital, *r* = 0.196	Low
Bahramnezhad et al. (34)	2017	Iran	201	≥65	70.02	Cross-sectional	Life satisfaction × friends network, *r* = 0.36	Moderate
							Life satisfaction × family network, *r* = 0.39	
							Life satisfaction × neighbors network, *r* = 0.20	
Au et al. (35)	2017	Hong Kong	365	65–74	68.91	Cross-sectional	Life satisfaction × social participation, *r* = 0.3	Low
							Life satisfaction × civic participation and employment, *r* = 0.09	
							Life satisfaction × community and health services, *r* = 0.31	
							Life satisfaction × social participation, *r* = 0.35	
			354	74–97	80.29		Life satisfaction × civic participation and employment, *r* = 0.35	
							Life satisfaction × community and health services, *r* = 0.34	

### Meta-analysis

**Figure 2:**
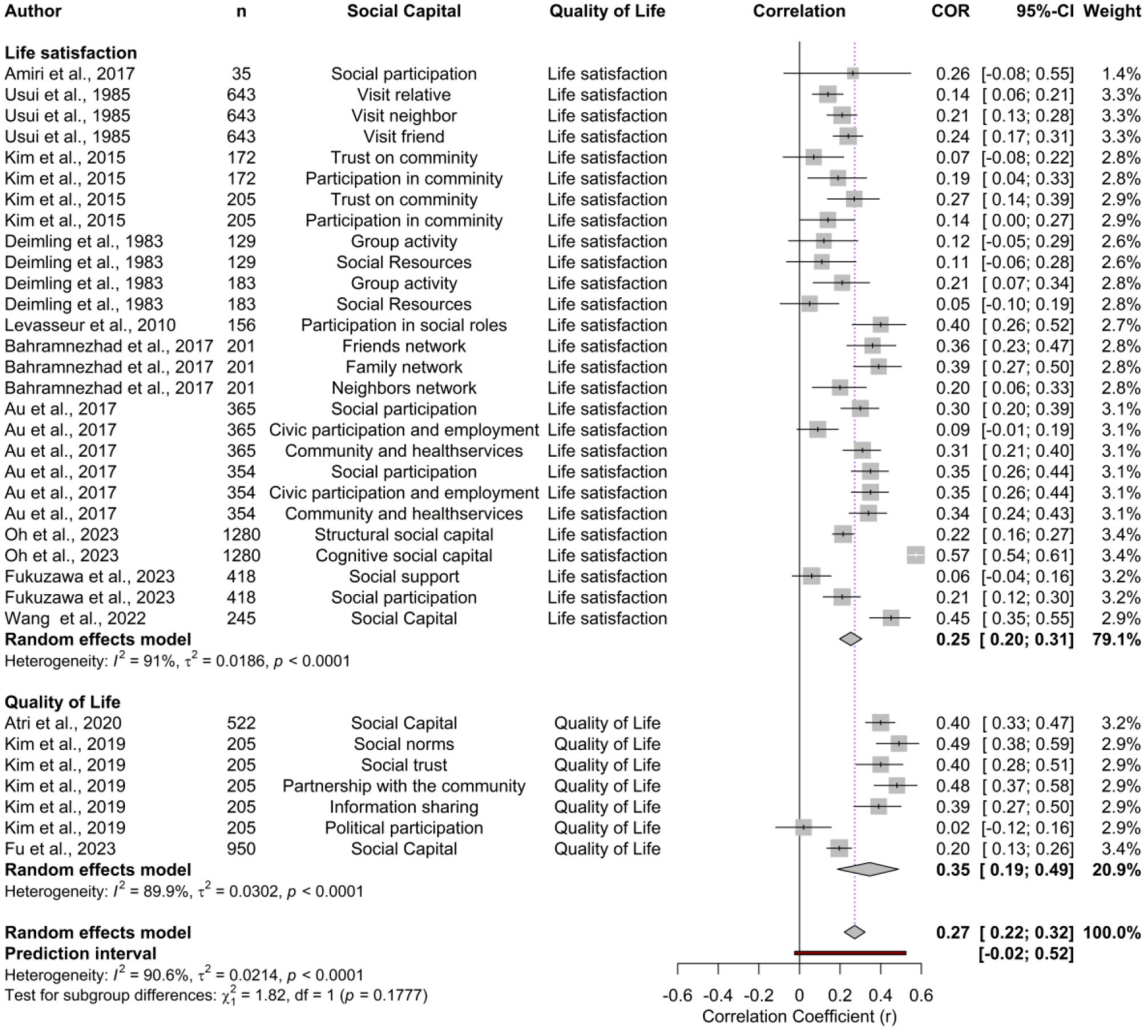
Forest plot of the association between social capital and quality of life.

**Figure 3:**
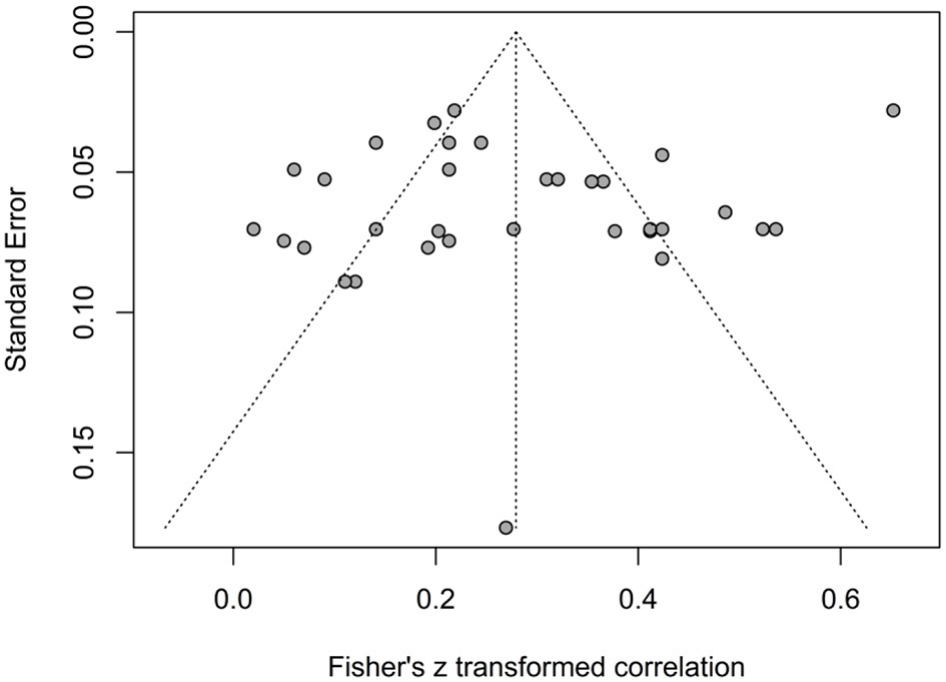
Funnel plot of the association between social capital and quality of life.

The correlation coefficient estimates reported by the individual studies ranged from 0.02 (95% CI: −0.12 to 0.16) to 0.57 (95% CI: 0.54–0.61). The forest plot illustrating the effect sizes for social capital and quality of life and life satisfaction is presented in Figure 2. Using a random-effects model, the overall pooled correlation across all studies was *r* = 0.27 (95% CI: 0.22–0.32 *p* < 0.001), reflecting a consistent positive association between social capital and quality of life in older adults. The prediction interval ranged from −0.02 to 0.52, suggesting that while most future studies are expected to find a positive association. Substantial heterogeneity was observed (*Q* = 350.61, τ^2^ = 0.0214, *I*^2^ = 90.6%, *p* < 0.001), indicating considerable variability across studies. The symmetrical distribution of points in the funnel plot (Figure 3), Egger test (*t* = −0.98, *p*-value = 0.3325) and Begg and Mazumdar test (*z* = 0.43, *p*-value = 0.6650) revealed no evidence of publication bias.

### Subgroup analyses

The subgroup meta-analyses are presented in Table 2. When stratified by the dimensions of social capital, cognitive social capital demonstrated a stronger association with quality of life, with a pooled effect size of 0.35 (95% CI: 0.18–0.49; *I*^2^ = 95.3) compared to structural social capital, which had a pooled effect size of 0.24 (95% CI: 0.19–0.29; *I*^2^ = 79.2), though the difference was not statistically significant (*p* = 0.1513). This suggests that factors such as trust, reciprocity, and perceived support may play a more important role in enhancing quality of life than structural elements like participation in organizations.

**Table 2:** Summary of subgroup analysis pooled effect sizes of the association between social capital and quality of life.

**Variables**	**No. of effect sizes**	**Pooled effect size, *r* (95% CI)**	**Prediction interval**	***I*^2^ %**	**Egger test**	**Begg test**	**Between groups (*p*-Value)**
Social capital	34	0.27 (0.22–0.32)	−0.02 to 0.52	90.6	0.3325	0.665	0.1513
Structural social capital	24	0.24 (0.19–0.29)	0.02–0.44	74.3			
Cognitive social capital	8	0.35 (0.18–0.49)	−0.15 to 0.70	95.3			
**Time period**							0.0966
Before 2015	22	0.24 (0.19–0.29)	0.04–0.42	68.9			
2015 and after	12	0.33 (0.22–0.44)	−0.10 to 0.66	95.5			
**By continent**							0.182
America	17	0.24 (0.16–0.32)	−0.08 to 0.51	81.5			
Asia	15	0.30 (0.23–0.37)	0.00–0.56	93.3			

In terms of quality-of-life measures, the pooled effect sizes were relatively consistent across life satisfaction and overall quality of life, with values of 0.25 (95% CI: 0.20–0.31; *I*^2^ = 91.0), and 0.35 (95% CI: 0.19–0.49; *I*^2^ = 89.9), respectively. Despite high heterogeneity, no significant differences were observed between these subgroups (*p* = 0.1777). This pooled correlation measure indicates that the beneficial impact of social capital is not limited to one specific outcome but applies broadly to older adults' perceived quality of life. When analyzed by time periods, studies published before 2015 reported a pooled effect size of 0.24 (95% CI: 0.19–0.29; *I*^2^ = 68.9), whereas those published in 2015 and after showed a stronger pooled effect size of 0.33 (95% CI: 0.22–0.44; *I*^2^ = 95.5). Furthermore, regional analysis revealed that studies from Asia exhibited a marginally greater pooled effect size (0.33, 95% CI: 0.23–0.37; *I*^2^ = 93.3) than studies from America (0.24, 95% CI: 0.16–0.32; *I*^2^ = 93.3), though the difference was again not statistically significant (*p* = 0.182). Although heterogeneity was high in the magnitude of the association across most subgroups (*I*^2^ > 70%), the overall direction of results was consistent: higher social capital is reliably associated with better quality of life among older adults, even if the strength of the association varies across contexts.

### Sensitivity analysis

The leave-one-out sensitivity analysis illustrated evaluated the robustness of the random-effects meta-analysis. The results demonstrated that eliminating any individual study did not significantly impact the overall pooled correlation coefficient. All pooled effect size values from omitting studies remained consistent within the range of 0.26 (95% CI: 0.21–0.31) to 0.28 (95% CI: 0.23–0.33). This analysis confirmed that the overall findings are stable and not unduly influenced by any single study, thereby reinforcing the robustness of the results.

### Meta-regression

The effect of study characteristics on the association between social capital and quality of life among older adults was investigated in a meta-regression analysis. The results showed that the year of publication (β = 0.0044, *p* = 0.0203) was a significant predictor suggesting that more recent research reported a somewhat larger impact of social capital on quality of life. This trend explained 13.79% of the between-study variability, indicating that time contributes to differences in effect sizes. However, factors including sample size, male ratio, and risk of bias evaluation exhibited no significant effect on the pooled effect size.

## Discussion

Our meta-analysis suggests that social capital is positively associated with quality of life and life satisfaction. Despite high variability, the consistent findings across different measures could suggest that promoting social capital could be an effective strategy for enhancing seniors' quality of life and life satisfaction. The findings will significantly influence public health programs and policies intended to improve the quality of life for aging adults across the globe. This aligns with previous studies highlighting the importance of relationships and community ties for wellbeing and quality of life (36, 37), as well as studies evaluating the effects of social prescribing interventions.

However, subgroup analysis showed cognitive social capital had a greater association than structural social capital, emphasizing the stronger influence of cognitive social dimensions (i.e., trust, reciprocity, shared values) than structural elements (i.e., network size). Our results are similar to a study conducted in Germany (38). Ferlander (36) similarly studied a sample in Sweden and found that cognitive social capital has a greater and consistent association than structural ties, to quality of life and happiness. Cognitive social capital reflects trust, belonging, and mutual support that directly enhances quality of life, while structural social capital refers to external networks that may not always bring real benefits (37). These findings suggest that prioritizing emotional and trust-based approaches to improve life satisfaction can be beneficial for old adults. These results imply that interventions should address the more emotional and psychological aspects of relationships that produce larger meaningful improvements in quality of life for older adults (e.g., programs addressing community trust). From a practical perspective, this means that interventions should not only encourage participation in groups but also strengthen feelings of trust, reciprocity, and belonging. Programs that focus on building safe and supportive community environments may therefore be more impactful than those that only expand social networks.

When considering specific outcomes, the association of social capital with quality of life appeared somewhat stronger (*r* = 0.35) than with life satisfaction (*r* = 0.25). Although this difference was not statistically significant, the trend suggests that social capital may influence broader assessments of wellbeing more strongly than evaluations of life satisfaction alone. One possible explanation is that quality of life measures typically captures multiple domains such as physical, psychological, and social functioning, where social capital can exert cumulative effects (39, 40). In contrast, life satisfaction reflects a more global judgment, which may be influenced by additional factors beyond health, psychology, or functioning status (41–43). These findings indicate that while social capital benefits both subjective and multidimensional wellbeing in older adults, its impact may be particularly pronounced in domains encompassed by quality of life assessments (37, 44).

Interestingly, studies published after 2015 had a stronger association between social capital and quality of life than studies published before 2015. This may reflect a growing awareness of social capital's relevance to aging populations, as well as improvements in measurement techniques over time (45–48). However, also changes in family relationships across cohorts now entering older ages may result in changes in risks of needing to provide care for others, widening gaps in the necessity of social support (49).

### Limitations

This meta-analysis has some limitations. Therefore, the findings of this study need to be interpreted with caution. A main limitation of the current meta-analysis is the high degree of heterogeneity across the published literature. Significant heterogeneity could be attributed to variations in study participants, study methodology, outcome assessment, or cultural context and can adversely affect the pooled effect size, thereby impacting the validity of the results. To overcome the heterogeneity problem, we used random effect meta-analyses models. Nonetheless, statistical analyses (20, 21), along with funnel plot assessments, indicated that there was no significant publication bias, which should strengthen the reliability of the current results. Second, the current meta-analysis only included peer-reviewed literature in English, which raises the possibility of selection bias due to the exclusion of valuable information from sources not in English or the gray literature. Third, we only used unadjusted measures of association (simple correlations) to limit causal inference, as these measures do not account for possible confounding variables that might mediate or confound the association. However, we performed a sensitivity analysis and detected no substantial fluctuation in the results, which supports the robustness of the findings.

## Conclusions

This meta-analysis demonstrates that higher levels of social capital are consistently associated with improved life satisfaction and quality of life in older adults. Cognitive social capital, encompassing trust, reciprocity, and shared values, emerged as a stronger predictor of wellbeing than structural social capital, such as network size or organizational participation. These findings suggest that interventions and policies should prioritize the quality of social relationships and trust-building efforts, rather than focusing solely on increasing the number of social ties.

At the same time, the substantial heterogeneity observed across studies reduces the certainty of the pooled estimates, highlighting the need for cautious interpretation. Future research should therefore aim to develop standardized tools for assessing social capital, conduct longitudinal studies to clarify causal mechanisms, and extend investigations to low- and middle-income countries, where evidence is scarce. Such steps would enhance comparability across studies and provide a stronger evidence base to guide effective interventions and policies for promoting healthy aging.

## Data Availability

The original contributions presented in the study are included in the article/Supplementary material, further inquiries can be directed to the corresponding author.
