# Supplementary material for: The association between social capital and quality of life in old adults: a systematic review and meta-analysis
Source: Front Public Health. 2025 Nov 6;13:1668696. doi: 10.3389/fpubh.2025.1668696 (PMC12629930; doi:10.3389/fpubh.2025.1668696)
Supplement: Supplementary file 1 [file Supplementary_file_1.doc]

Supplementary information for the Article:

The Association between Social Capital and Quality of life in the Elderly: A Systematic Review and meta-analysis

**Contents**:

1. **Table S1:** Strings for literature search for Association between Social Capital and Quality of Life in Older Adults
2. **Table S2**: Newcastle-Ottawa Scale Quality Assessment of the included studies.

**Table S1:** Strings for literature search for Association between Social Capital and Quality of Life in Older Adults

| **PubMed**  (n=1685) | (“old people”[Title/Abstract] OR “old adult*”[Title/Abstract] OR “old age”[Title/Abstract] OR “older people”[Title/Abstract] OR “older adult*”[Title/Abstract] OR “older age”[Title/Abstract] OR “geriatric”[Title/Abstract] OR “elder*”[Title/Abstract] OR “senior*”[Title/Abstract] OR older person*[Title/Abstract] OR old person*[Title/Abstract] OR aging adult*[Title/Abstract] OR aging person*[Title/Abstract] OR ageing adult*[Title/Abstract] OR ageing person*[Title/Abstract] OR geriatrics[Title/Abstract] OR “aged”[Mesh]) AND (“quality of life”[Title/Abstract] OR “well-being”[Title/Abstract] OR “life satisfaction”[Title/Abstract] OR “QoL”[Title/Abstract] OR “life quality”[Title/Abstract] OR “quality of life”[Mesh]) AND (“social capital”[Title/Abstract] OR “community networks”[Title/Abstract] OR “social support”[Title/Abstract] OR “social cohesion”[Title/Abstract] OR “social resources”[Title/Abstract]). |
| --- | --- |
| **PsycINFO**  (n=359)  **and CINAHL**  (n = 294) | AB (old people or old adult* or old age or older people or older adult or older age or geriatrics or elder* or senior or older person* or old person* or aging adult* or aging person* or ageing adult* or ageing person* or geriatrics or senior* or aged) AND TI (quality of life or QoL or well-being or life quality or life satisfaction) AND TI (social capital or community networks or social support or social cohesion or social resources). |

**Table S2**: Newcastle-Ottawa Scale Quality Assessment of the included studies.

| CROSS-SECTIONAL STUDIES | Selection | | | | Comparability** | Outcomes | | TOTAL (maximum 10✯) | **Risk of Bais** |
| --- | --- | --- | --- | --- | --- | --- | --- | --- | --- |
|  | Representativeness of sample | Sample Size | Non-respondents | Ascertainment of the exposure (risk factor) ** |  | Assessment of outcomes** | Statistical test |  |  |
| Amiri et al., 2017 | – | – | – | ****** | - | ** | * | 5 | Moderate |
| Oh et al., 2023 | ***** | ***** | **-** | ****** | ****** | ** | ***** | 9 | Low |
| Usui et al., 1985 | ***** | ***** | **-** | ***** | ****** | ** | ***** | 9 | Low |
| Fukuzawa et al., 2023 | * |  | * | ** | ** | ** | * | 9 | Low |
| Kim et al., 2015 | * | – | * | ** | ** | ** | * | 9 | Low |
| Atri et al., 2020 | * |  | * | ** | ** | ** | * | 9 | Low |
| Deimling et al., 1983 | * | * | * | ** | ** | ** | * | 10 | Low |
| Kim et al., 2019 | - |  | * | ** | ** | ** | * | 8 | Low |
| Levasseur et al., 2010 | - |  | * | ** | ** | ** | * | 8 | Low |
| Wang et al., 2022 |  | * | * | ** | ** | ** | * | 9 | Low |
| Fu et al., 2023 | * | * | - | ** | ** | ** | * | 9 | Low |
| Bahramnezhad et al., 2017 | - | * | - | ** | - | ** | * | 6 | Moderate |
| Au et al., 2017 | - | * | - | ** | ** | ** | * | 8 | Low |
